# Cognitive-Enhancing Effects of Bioactive Compounds and Traditional Herbal Medicines in Elderly Patients with Metabolic Syndrome

**DOI:** 10.3390/biom16040535

**Published:** 2026-04-03

**Authors:** Pouria Sefidmooye Azar, Shiva Akhlaghi, Zia Shariat-Madar, Fakhri Mahdi

**Affiliations:** 1 Department of Nutrition & Family Sciences, University of Central Arkansas, Conway, AR 72035, USA; pazar@uca.edu; 2 Division of Medicinal Chemistry, School of Pharmacy, University of Mississippi, University, MS 38677, USA; sakhlagh@go.olemiss.edu; 3 Division of Pharmacology, School of Pharmacy, University of Mississippi, University, MS 38677, USA; madar@olemiss.edu

**Keywords:** insulin resistance, oxidative stress, cellular senescence, vascular complications, vascular dementia, channelopathies, adipose tissue dysfunction, inflammatory pathways, hepatic steatosis, skeletal muscle insulin resistance

## Abstract

Aging is a multifactorial process characterized by progressive physiological changes, including cellular senescence, cellular loss, and organ decline, which collectively accelerate the development of metabolic syndrome (MetS) in older adults. MetS, in turn, not only significantly increases the risk of cardiovascular disease (CVD) but also contributes to decreased functional and cognitive capacity, partly due to diminished ability to adapt to metabolic stress. While genetic predisposition has a substantial influence on the risk of developing MetS, other intrinsic factors, including chronic inflammation, insulin resistance (InsR), and altered neurohormonal activation, also play crucial roles. Targeted therapies, lifestyle interventions, and pharmacotherapy can decelerate the progression of CVD, improving the likelihood of survival with favorable neurological and functional outcomes in older individuals with MetS. However, adverse drug reactions and the lack of adequate interventions for cognitive decline have led to the emergence of self-medication with nonprescription products. The anti-inflammatory, antioxidant, anti-channelopathy, antiaging, and neuroprotective properties of flavonoids, alkaloids, polysaccharides, and polyphenols found in key traditional medicines have shown promising potential in the treatment of MetS-induced cognitive decline. This narrative review summarizes current evidence on bioactive compounds and herbal medicines that may offer cognitive benefits in elderly patients with MetS.

## 1. Introduction

The older population has been growing globally from 1960 to 2024, although at varying levels, according to recent reports published by the United Nations (UN) Population Division [1] and the United States Census Bureau [2]. The global number of people aged 65 years and older is projected to double within the next thirty years.

Older adults are at increased risk of developing multiple chronic metabolic diseases, which are strongly associated with cognitive decline [3,4]. Poorly controlled metabolic diseases and longer disease duration significantly elevate illness risk and are major drivers of vascular cognitive impairment [5]. These conditions disrupt the structural and functional integrity of the cerebral microcirculation [6], promoting microvascular rarefaction [7] and contributing to both micro- and macro-vascular dysfunction, as well as impaired neurovascular coupling [8]. Together, these alterations lead to dysregulated cerebral blood flow and nutrient delivery, resulting in neuronal injury and accelerated cognitive decline. Chronic metabolic diseases also compromise blood–brain barrier integrity, triggering neuroinflammation [9], a key contributor to cognitive deterioration.

Cognitive impairment in individuals with metabolic diseases is a multifaceted phenomenon and a well-recognized contributor to poor health outcomes among the global elderly population, influencing how the body utilizes essential metabolites and transitions from a healthy, well-functioning state to a chronic and debilitating disease.

This review critically delves into the pathophysiology and clinical significance of metabolic disease-induced comorbidities and emphasizes their role in cognitive decline, with the aim of consolidating current understanding of how these conditions are linked to the most common cause of cognitive impairment. It explores the significant role of metabolic diseases in the context of fading cognitive function, highlighting current knowledge on how metabolic disorders affect cognitive function and the associated challenges. We intend to offer a roadmap for identifying research gaps and outlining opportunities for potential clinical translation.

In this narrative review, PubMed, Scopus, Google, and Google Scholar were searched in tandem to identify core mechanisms involved in MetS and herbal medicines used to improve their functionality. Google was included because of its broad search capabilities for locating various document types, such as conference papers, patents, government reports, and statistics. The literature search was carried out from 2020 to 2025 and included combinations of keywords as follows: “herbal medicine”, “herbal supplements”, “traditional medicine”, “bioactive compounds”, “natural products”, “MetS”, “InsR”, “cognitive decline”, “aging”, “hallmarks of aging”, “oxidative stress”, “mitochondrial dysfunction”, “vascular dementia”, “inflammation”, and “hepatic inflammation”. Inclusion criteria were peer-reviewed articles published in English between 2020 and 2026. Research articles were also selected based on their relevance to the scope of this review. Exclusion criteria included non-English articles, conference abstracts without full text, editorials, duplicate studies, and publications irrelevant to the objectives of this review.

This review also summarizes a thorough grasp of the current treatment landscape of traditional medicines for metabolic disease-mediated cognitive decline, evaluates their emerging global roles, and highlights their evidence-based integration approaches, including potential molecules derived from traditional medicines that are currently undergoing clinical trials. The review specifically aims to provide a comparative analysis of representative traditional medicines from different regions that show promise in treating metabolic disease-mediated cognitive deterioration.

## 2. The Complex Interplay Between the Individual Components of the MetS

MetS is an umbrella term encompassing a diverse yet interconnected set of metabolic dysregulations that exhibit spatial and temporal variability and progress silently across multiple organ systems. The severity of MetS is influenced by numerous intrinsic and modifiable factors.

MetS is commonly observed not only in individuals with central obesity and reduced high-density lipoprotein (HDL) levels but also in those presenting with any two of the following metabolic abnormalities: hypertriglyceridemia, hyperglycemia, and high blood-angiotensin II (Ang II). This highlights the significant roles of triglycerides [10], insulin [11], and angiotensin-related molecules [12] in their respective target tissues. Each of these components and the potential role of ion channels in the development of InsR, impaired insulin signaling, and inflammation are discussed in the following sections.

### 2.1. Insulin Is a Major Metabolic Hormone

**Figure 1:**
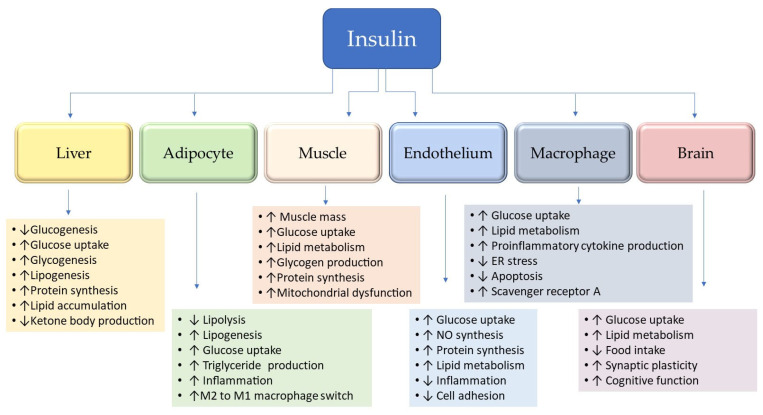
Schematic presentation of insulin action on multiple tissues.

Insulin, a key metabolic hormone, regulates glucose distribution, lipid metabolism, and protein synthesis through complex networks of both positive and negative feedback mechanisms (Figure 1). Most importantly, insulin plays a key role in cognitive processes, and the inability of insulin to regulate glucose metabolism in insulin-resistant older adults results in cortical hypoperfusion and hypometabolism, leading to diverse cognitive impairments. These tightly controlled processes are essential for preserving the functional integrity of diverse tissues and organs.

Insulin exerts its effects through interactions with insulin receptors (IRs), which can be nuclear-associated [13,14] or act through classical phosphorylation-dependent signaling pathways [14]. IRs exist in two primary isoforms, A and B, which exhibit similar affinities for insulin but differ in their binding affinities for insulin-like growth factor 1 (IGF-1) and insulin-like growth factor 2 (IGF-2) [15,16].

Insulin functions not only as a vasodilator but also as an anti-inflammatory agent. These effects are mediated in part through nitric oxide (NO) release and inhibition of the transcription factor nuclear factor-κB (NF-κB). Impaired insulin signaling disrupts cellular responses through dysregulation of the mitogen-activated protein kinase (MAPK) and phosphatidylinositol 3-kinase (PI3K) pathways, both of which relay signals through the tyrosine phosphorylation of insulin receptor substrates 1 and 2 (IRS-1 and IRS-2), ubiquitously expressed proteins in human tissues.

IRS-1 is primarily involved in development, growth, and peripheral insulin responsiveness, whereas IRS-2 plays a key role in cerebral growth, body weight regulation, and glucose homeostasis [17]. Upon phosphorylation, IRS-1 and IRS-2 activate the PI3K/Akt pathway, thus promoting the initiation of insulin-mediated metabolic processes.

### 2.2. Key Insulin-Target Tissues

InsR tissues are thought to contribute to enhanced inflammatory signaling, dysregulation of hormonal factors, activation of endoplasmic reticulum (ER) stress pathways, and the accumulation of excess lipids in organs and tissues. Most of the mechanisms associated with InsR and pancreatic β-cell dysfunction are relatively well understood. However, the mechanisms underlying communication between insulin and insulin-responsive target tissues are far less well characterized. Current evidence suggests that InsR develops early in the disease course and is accompanied by abnormal responses in insulin-responsive target tissues.

Over time, a combination of persistent InsR and progressive pancreatic β-cell dysfunction leads to insufficient insulin secretion in the presence of hyperglycemia. In response, insulin-responsive target tissues undergo physiological adaptations, including activation of inflammatory pathways, increased oxidative stress, and enhanced mobilization and release of fatty acids as an alternative energy source when glucose utilization is impaired.

#### 2.2.1. Hepatic Tissue InsR

InsR, obesity, and dyslipidemia are associated with metabolic dysfunction-associated steatotic liver disease (MASLD) [18], a major cause of liver-related morbidity. MASLD includes hepatic steatosis, previously known as fatty liver infiltration [19]. It affects more than a quarter of the global population [18], and half of the global adult population is predicted to develop MASLD by 2040 [20]. MASH is considered a progressive form of fatty liver disease [21] with a bidirectional association with metabolic dysfunction [22].

If left unresolved, MASLD can progress to hepatic inflammation and fibrosis, eventually leading to cirrhosis [23], a phenomenon known as the transition from MASLD to metabolic dysfunction-associated steatohepatitis (MASH), the more advanced stage of the disease. MASH is mainly mediated by inflammation, oxidative stress, and dysbiosis [23], and it is associated with impaired glucose metabolism, hypertension, and atherogenic dyslipidemia [18].

#### 2.2.2. Adipose Tissue InsR

Adipose tissue regulates systemic energy metabolism not only through adipokine production but also via energy storage, which is fundamental for ensuring the energy supply to other organs, such as skeletal muscles and the liver, during the fasted state. Dysfunction of adipose tissue contributes to a plethora of metabolic disorders, influencing several systems in parallel. Accordingly, the relationship between adipocytes and insulin-mediated receptor activation remains important [24]. This relationship is frequently examined to better understand the molecular interactions and physiological consequences that arise in the absence of InsR.

Insulin plays fundamental roles in the normal physiology of adipose tissue via IRs, namely, IR–A and IR–B. Dysfunctional adipose tissue releases cytokines that impair insulin signaling pathways, contributing to InsR. Interestingly, alterations in the IR–A:IR–B ratio appear to contribute to metabolic abnormalities, such as lipid metabolism and redistribution, during the onset of InsR [25].

#### 2.2.3. Skeletal Muscle InsR

Skeletal muscle plays a key role in whole-body glycemic control, a process that relies on insulin signaling and the presence of functional glucose transporters within muscle tissue [26].

During insulin stimulation, increased muscle capillary perfusion enhances the delivery of both insulin and glucose to skeletal muscle [27], maintaining interstitial glucose concentrations and supporting overall glucose uptake. Evidence indicates that activation of genes such as sirtuin (*SIRT*) 2 and F-Box and WD Repeat domain-containing 5 (FBXW5), which regulate lipid metabolism, autophagy, and mechanistic target of rapamycin (mTOR) signaling, has been correlated with insulin sensitivity in muscle [28].

Desensitization of muscle tissue to chronically elevated blood glucose levels contributes to InsR and exacerbates skeletal muscle atrophy and dysfunction [29]. Evidence suggests that skeletal muscle InsR develops prior to the onset of β-cell failure and the development of symptomatic T2DM [30]. In InsR, insulin-stimulated glucose uptake in skeletal muscle is impaired. Notably, skeletal muscle InsR and IR signatures have been identified in both normoglycemic individuals and those with T2DM [31,32], supporting the concept that muscle InsR is an initiating factor in the pathogenesis of T2DM. As skeletal muscle is the primary site of insulin-stimulated glucose uptake in humans, it is recognized as a major contributor to systemic InsR [32]. Reduced skeletal muscle mass has been identified as a predictor of InsR progression in older adults [33]. Dysfunctional skeletal muscles contribute to the development and progression of MetS, including obesity, T2DM, and sarcopenia, through impaired endocrine and metabolic functions.

### 2.3. Hypertension and T2DM Coexist as Part of MetS

**Figure 2:**
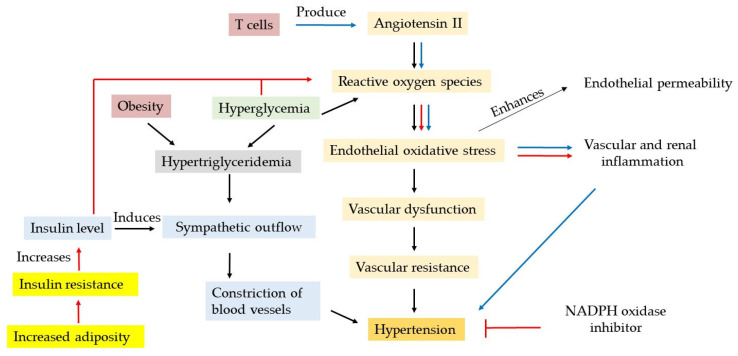
A schematic representation of the interrelationship among components of MetS leading to endothelial oxidative stress, increased endothelial permeability, vascular resistance, and vascular and renal inflammation.

Ang II, a circulating hormone, is the final bioactive metabolite of the renin–angiotensin system (RAS). The RAS regulates a wide range of biological functions, including blood pressure maintenance, electrolyte homeostasis, and fluid balance, thus ensuring adequate blood flow to organs and tissues. The RAS exists in two forms, a circulating RAS and a tissue-specific RAS, which is expressed in organs such as adipose tissue, the brain, and T cells (Figure 2). Dysregulation of tissue RAS expression or elevated systemic Ang II levels contributes to multiple pathological conditions, including CVD, T2DM, and renal fibrosis [34].

Elevated circulating Ang II levels, T cell-derived Ang II, or hyperglycemia enhances the production of reactive oxygen species (ROS), leading to cytosolic oxidative stress in endothelial cells through reduced NO bioavailability [35]. Vascular remodeling, driven by persistent Ang II-induced oxidative stress [36], contributes to the onset and progression of increased vascular resistance and promotes hypertension, a key component of cardiometabolic syndrome. Other MetS components, such as hyperglycemia- and obesity-induced hypertriglyceridemia, further increase sympathetic outflow, resulting in vasoconstriction and elevated blood pressure. In addition, adiposity-associated InsR promotes compensatory insulin secretion, leading to hyperinsulinemia, which can induce hypertension via sympathetic activation and further exacerbate ROS production [37]. Taken together, hyperglycemia and dyslipidemia play integral roles in the development of vascular dysfunction and hypertension.

Ang II, through both local and systemic actions, can induce inflammation in critically ill patients regardless of age. It promotes oxidative stress and activates immune cells upon binding to Ang II-responsive tissues [38,39], contributing to vascular and renal inflammation (Figure 2).

Moreover, evidence indicates that upregulation of Ang II type 1 receptor (AT1R) expression in the liver leads to dysregulated glucose and lipid metabolism and increased hepatic lipid accumulation [40]. Polymorphisms in genes encoding components of the RAS are associated with increased susceptibility to MetS [41]. Consistently, hypertensive transgenic mice expressing the human AT1R haplotype are prone to developing MetS [42]. Collectively, hyperglycemia and dyslipidemia appear to interact with elevated Ang II-induced hypertension to promote vascular injury, which can further exacerbate hypertensive pathology.

### 2.4. Several Competing Theories Have Been Proposed to Explain the Underlying Mechanisms of MetS

- InsR contributes to metabolic dysfunction-associated alterations in adipose tissue size and function. Reduced insulin action limits fatty acid uptake in adipose tissue, leading to tissue remodeling characterized by immune cell infiltration, chronic low-grade inflammation, and altered secretion of adipokines. These changes have led to the hypothesis that they promote the development and progression of metabolic disorders, including T2DM, hypertension, and ectopic fat accumulation, conditions that commonly coexist and synergistically drive the progression of MetS.
- Chronic activation of RAS promotes vascular endothelial dysfunction, resulting in fibrosis, increased ROS production, reduced NO availability, increased inflammatory response, and elevated sympathetic nervous system (SNS) activity. These alterations are proposed to contribute to the development of not only T2DM but also MetS.
- Loss of buffering capacity and increased stiffness of the macrovasculature reduce the delivery of steady blood flow to the microvasculature, impairing insulin-mediated glucose disposal and promoting dysregulated inflammatory and oxidative responses. Recent intensive investigations into macrovascular atherosclerosis and microvascular endothelial dysfunction across tissues have advanced understanding of T2DM and hypertension pathogenesis, and they propose that these processes contribute to the development of MetS.
- Overaction of SNS, driven by metabolic abnormalities such as obesity, impaired baroreflex sensitivity, hyperinsulinemia, and elevated adipokine levels, promotes a decline in insulin sensitivity and can accelerate the development of central obesity, InsR, and cardiovascular risk. These changes are also proposed to contribute to the development of MetS.
- Emerging research has revealed a complex interplay between genetic predisposition and environmental factors, particularly in the context of metabolic diseases. Obesity, T2DM, hypertension, and InsR are widely recognized as heritable, indicating a genetic predisposition, and several candidate genes have been postulated in the etiology of these conditions. It has been proposed that lifestyle choices and environmental exposures may play a pivotal role in mitigating obesity, T2DM, hypertension, and InsR through a healthy diet, reduced sedentary behavior, and avoidance of harmful habits.

There is an association between MetS and sustained insulin-responsive tissue damage, a concept that has gained considerable attention among investigators in the field. It remains unclear, however, whether changes in insulin-responsive tissues precede the development of MetS or primarily result from prolonged elevations in blood glucose, blood pressure, overnutrition, or hormonal dysregulation.

Obesity, T2DM, hypertension, and dyslipidemia, which cluster to form MetS, share dysregulated inflammatory and oxidative responses that play key roles in the pathogenesis of these conditions. Over the past three decades, increasing efforts have focused on identifying plants and herbal medicines rich in specific classes of bioactive compounds that can scavenge ROS, enhance NO bioavailability, boost cellular antioxidant capacity, and reduce inflammatory mediators. These effects may improve insulin sensitivity, support weight management, and exert antidiabetic, antihypertensive, and lipid-lowering actions. While some of these medicinal plants have been validated, others remain inconclusive or have been disproven.

### 2.5. Vascular Cognitive Impairment and Vascular Dementia

While vascular cognitive impairment and vascular dementia can occur following a stroke, they may also result from damaged blood vessels and reduced circulation [43]. Endothelial-related diseases and macro- and microvasculature dysfunction are characterized by decreased arterial wall distensibility, vascular calcification, and increased arterial wall thickness. These structural and functional abnormalities—when augmented by a cluster of metabolic disorders, including hyperglycemia; prolonged elevation of Ang II, which can contribute to sustained hypertension; reduced circulating myokines; and dyslipidemia—disrupt vascular homeostasis. Consequently, elderly individuals face a significantly higher risk of vascular injury, which can manifest as heart disease, atrial fibrillation, venous thromboembolism, stroke, and organ damage, as well as cognitive decline involving progressive losses in memory, reasoning, and attention. This type of vascular cognitive impairment, known as subcortical ischemic vascular dementia, results from damage to the small blood vessels and nerve fibers within the brain’s white matter.

Endothelial dysfunction has emerged as a key contributor to the etiology of MetS, affecting various signaling pathways critical for physiological processes such as blood flow, inflammation, cell adhesion, and coagulation. Diabetes [44], hypertension [45,46], and potentially MetS [47] are major drivers of both microvasculature and macrovasculature complications, which share common risk factors and exhibit a bidirectional relationship.

Crosstalk between dysfunctional microvascular and macrovascular systems [48], along with subsequent structural vascular damage, reduces blood flow and nutrient delivery, including impaired pancreatic microcirculation and diminished glucose delivery to target organs. Elderly individuals with a combination of these conditions are at a significantly higher risk of cognitive decline.

## 3. Cognitive Deterioration in the Context of Metabolic Diseases

**Figure 3:**
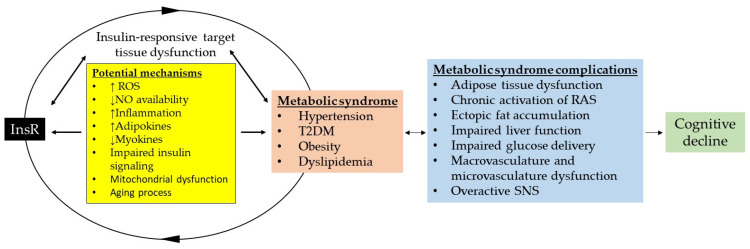
The feedback cycle between InsR and MetS contributes to an increased risk of vascular cognitive impairment and dementia.

MetS affects a reported 14.5–31% of women and 9–25.7% of men according to a systematic review and Bayesian modelling of data points involving 45,549,151 adults [49]. Patients with MetS are at an increased risk of damage to organs, including the brain. There is a direct correlation between MetS and obesity, chronic activation of RAS, MASLD, inflammation, dyslipidemia, T2DM, InsR, hypertension, impaired glucose delivery, overactive SNS, and potential cognitive decline (Figure 3). We briefly describe a bidirectional relationship that exists between the InsR and the components of MetS that influence insulin-responsive target tissues, leading to the activation of multiple signaling pathways.

It is well established that obesity is an unusually heterogeneous medical condition [50]. Although obesity is associated with several life-threatening comorbidities [51], not all individuals with obesity develop these conditions. The combined effects of body fat distribution and abnormal remodeling of adipose tissue are major correlates of perturbed physiological pathways, leading to dysfunctions in the depot, characterized by hypertrophy of adipocytes [52], a vital contributor to InsR.

Both hepatic and adipocyte InsR perpetuate dyslipidemia and hyperglycemia. A decline in muscle mass due to InsR impairs glycogen production and reduces the secretion of myokines, leading to decreased insulin sensitivity. It has been reported that myokines not only regulate glucose and lipid metabolism but also improve glucose disposal [53]. Consequently, dysfunctional responses in these insulin-target organs can not only trigger failure in downstream organs in a domino-like effect but also reflect and exacerbate systemic conditions that predispose individuals to hypertension, diabetes, and MetS, a phenomenon that becomes more pronounced with aging [49] (Figure 3).

Notably, both T2DM [54] and hypertension [55] significantly contribute to macrovasculature and microvasculature complications, including ischemic heart disease, peripheral vascular disease, cerebrovascular disease, and retinopathy, as well as increased inflammatory response and excess oxidative stress [56], often involving InsR. Hypertensive patients with T2DM have higher rates of stroke compared to healthy individuals, independent of risk factors. Insulin-resistant older adults exhibit deterioration of cognitive function. The association between InsR and conditions such as T2DM, hypertension, or aging is unquestionable.

There is a need for new treatments that promote microvascular health in order to delay the pathogenesis of vascular cognitive impairment in patients with MetS. Notably, the potential therapeutic benefits of herbal medicine for the treatment of vascular-induced cognitive decline have been investigated over the past decade. For instance, clinical studies have shown strong evidence that SaiLuoTong (SLT) not only has the potential to improve neurocognition in healthy individuals but also significantly improves cognitive function in patients diagnosed with vascular dementia [57,58]. Collectively, a multidisciplinary MetS management strategy appears to be needed to prevent the development of vascular cognitive impairment. In this review, we explore and summarize the potential therapeutic benefits of anti-aging of bioactive compounds and herbal medicines that show promising cognitive benefits for elderly patients with MetS from 2020 to date.

## 4. Ion Channels Play an Important Role in the Development and Progression of MetS

### 4.1. Potassium Channels

**Figure 4:**
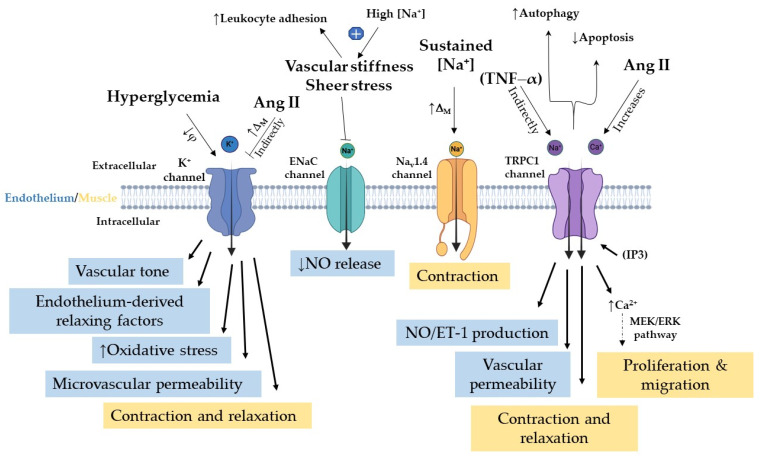
This figure depicts how different vascular cation channel networks contribute to vascular homeostasis through different mechanisms. Channel images were created using BioRender. Akhlaghi, S. https://app.biorender.com/illustrations/6969c48d00f26499747783a7 (accessed on 21 February 2026).

Potassium ion (K^+^) channels serve not only as key regulators of vascular smooth muscle cell membrane potential but also help maintain cardiac rhythm stability. They are therefore important determinants of vascular smooth muscle contractility, vascular tone, and myocardial electrical activity. Both vascular endothelial cells and vascular smooth muscle cells express several types of K^+^ channels [59], which contribute to vascular homeostasis through different mechanisms (Figure 4).

Arterial tension is closely linked to membrane potential and the activity of ion channels within the vascular wall. In vascular smooth muscle cells, K^+^ channels play a key role in regulating contractility and vasoconstriction/vasodilation by controlling membrane potential and directly influencing intracellular calcium levels [60]. In contrast, endothelial K^+^ channels regulate vascular tone indirectly by modulating the release of endothelium-derived relaxing factors and contributing to the regulation of microvascular permeability.

Activation of K^+^ channels in endothelial cells supports the maintenance of vascular tone and blood flow [61], while in vascular smooth muscle cells, these channels govern both contraction and relaxation. Dysfunction of the vascular K^+^ channel network can therefore significantly alter arterial tone.

Ang II induces smooth muscle contraction in blood vessels, leading to increased vascular resistance and elevated blood pressure [62]. Notably, Ang II modulates vascular tone by influencing multiple ion channels, including K^+^ channels [63]. Inhibition of K^+^ channel activity by Ang II leads to membrane depolarization (Δ_M_), which promotes contraction and reduces blood flow (Figure 4). Hyperglycemia also affects K^+^ channel function through oxidative stress-mediated mechanisms [64,65]. Hypertension also impairs K^+^ channel activity, leading to reduced vasodilatory capacity (φ) (Figure 4) [66,67]. Dysfunction of these channels contributes to vascular disease and hypertension by disrupting the regulation of basal vascular tone, ultimately impairing blood flow and increasing cardiovascular risk. For a detailed overview of the roles of K^+^ channels, readers are referred to previous reviews [68], including their interactions with perivascular adipose tissue and their involvement in diabetes mellitus and its complications [69].

### 4.2. Sodium Channels

Vascular endothelial cells play a significant role in sodium homeostasis. The epithelial sodium channel (ENaC) is expressed in vascular tissue (Figure 4) [70,71] and skeletal muscle [72]. Skeletal muscle expresses Na_v_1.4, a voltage-gated sodium channel that triggers muscle contraction by initiating and propagating action potential (Figure 4) [73]. This condition is caused by sustained sodium conductance, which is associated with hyperkalemia. Mutations of Na_v_1.4 appear to cause muscle stiffness, which is known as myotonia [73]. ENaC and Na_v_1.4 play important roles in endothelial and skeletal muscle function, and their dysfunction can contribute to metabolic disease.

In cultured endothelial cells, ENaC acts as a negative modulator of endothelial eNOS and NO production in resistance arteries [74]. Evidence indicates that the vasodilatory response to shear stress is enhanced by ENaC blockade [74]. The study shows that ENaC activation reduces NO release, whereas its inhibition results in elevated NO release and vasodilation. Further investigations are needed to confirm this paradigm. Deletion of ENaC prevents stiffening of the endothelial cells both in vitro and in vivo [75]. Genetic deletion of endothelial ENaC alters the vascular response to fluid shear [76]. Arterial stiffness is an independent risk factor for CVDs [77]. ENaC contributes to diabetes, hypertension, and aging, leading to impaired IR signaling, a key factor in MetS.

Patients with diabetes [78], hypertension [79], or chronic kidney disease [80,81] exhibit damage to the endothelial surface layer. Vascular stiffening is an age-related phenomenon that is accelerated by InsR, particularly in the context of diabetes. A key downstream mediator of IR activation is the endothelial Na^+^ channel, which plays an important role in endothelial dysfunction, cardiovascular fibrosis, and vascular stiffening [82]. Incubation of endothelial cells with high sodium concentrations leads not only to increased stiffness of the endothelial surface layer but also to a significant decrease in endothelial surface-layer heparan sulfates (a key component of the endothelial glycocalyx), resulting in reduced sodium buffering capacity (↓φ) (Figure 4) [83]. The endothelial glycocalyx binds sodium through its negatively charged surface, thereby protecting endothelial cell function and regulating vascular permeability via its buffering effect [84].

These alterations impair barrier function, facilitating sodium entry into endothelial cells, either directly or through increased activity of endothelial sodium channels [85]. Sodium-induced increases in endothelial stiffness and reduced buffering capacity enhance leukocyte adhesion via both vascular cell adhesion molecule 1 (VCAM-1)-dependent and intracellular adhesion molecule 1 (ICAM-1)-dependent mechanisms [86]. These processes play a significant role in regulating homeostasis and in pathologic states, including T2DM [87], Ang II-induced arterial hypertension and vascular dysfunction [88], and in cognitive impairment pathogenesis [89]. Additionally, these changes reduce shear stress-mediated NO production [90] and increase ROS levels, leading to vascular remodeling that drives impaired vascular homeostasis and subsequent dysregulation of blood pressure [91].

Non-voltage-dependent Na^+^ channels, such as the Na^+^ leak channel (NALCN), are predominantly expressed in neurons [92] but also appear to contribute to the resting membrane potential of arterial smooth muscle cells, where they help limit excessive depolarization. Mutations in NALCN are associated with cognitive delay [93]. NALCN-mediated Na^+^ influx influences membrane excitability [94] and interacts with sodium-activated potassium (K^+^) channels [95].

It has been proposed that sodium-activated potassium currents help lower blood pressure [95], particularly during heightened SNS activity [96], which increases heart rate and blood pressure to enhance blood flow. Mutant animals lacking normal NALCN function are more vulnerable to vasoconstrictive agents, resulting in a paroxysmal hypertensive phenotype. However, evidence also suggests that sodium-activated potassium currents oppose arterial smooth muscle depolarization independently of a specific relationship with the SNS [95]. Taken together, these studies suggest that endothelial/muscle sodium transporters are able to alter vascular signaling.

### 4.3. Transient Receptor Potential (TRP) Isoforms in MetS

Endothelial cells express several TRP channel isoforms, each exhibiting different functions and expression profiles (Figure 4) [97]. TRP channels, cation-permeable channels, play essential roles in numerous tissues [98]. Among TRP channels, transient receptor potential canonical 1 (TRPC1) channels contribute to endothelial-dependent vasodilation [99] and vascular permeability, as demonstrated in TRPC1/TRPC4 double knockout mice [100]. Evidence indicates that TRPC channels assemble as either homo- or heterotetramers and play a crucial role in regulating intracellular calcium [101]. Another study provided evidence that TRPC1 may serve as a negative regulator for TRPC4 and TRPC5, thus protecting neurons from cell death via reducing calcium influx [102]. TRPC1, a nonselective cation channel, is expressed in vascular endothelial cells [103], the cerebellar hemisphere [102], and skeletal muscles [102], as well as in adipocytes, where it appears to promote increased autophagy and reduced apoptosis (Figure 4) [104]. A later study demonstrated that TRPC1 knockout mice display reduced adipocyte content in both subcutaneous and visceral adipose tissues, underscoring the importance of TRPC1 in adipocyte regulation [104].

TRPC1 channels are activated in a diacylglycerol (DAG)-, inositol trisphosphate (IP3)-, or phospholipase C (PLC)-independent manner; however, TRPC channels primarily support calcium and sodium influx in response to agonists of G protein-coupled receptors (GPCRs) [105]. TRPC1 has also been implicated in the regulation of endothelial repair capacity [106]. Notably, TRPC1 expression is increased under high-glucose milieu compared with other TRPC isoforms, highlighting its key role in vascular homeostasis [107]. Enhanced TRPC1-dependent calcium entry has been proposed as a key driver of cellular dysfunction in this context [107,108]. Surprisingly, TRPC1 knockout mice exhibit normal vasculature, indicating that targeted TRPC1 silencing may be well tolerated. Therefore, targeted inhibition of TRPC1 in vascular endothelial cells may represent a viable therapeutic strategy to alleviate hyperglycemia-induced endothelial dysfunction.

However, genetic ablation of TRPC1 in endothelial cells promotes the formation of heteromeric TRPV4–TRPP2 channels [105], which have been associated with reduced cardiac hypertrophy [109]. Moreover, a non-synonymous single-nucleotide polymorphism (rs7638459) in the TRPC1 (*TRPC1*-rs7638459) gene has been identified as an independent risk factor for T2DM in the Han Chinese population [110]. Thus, it is reasonable to suggest that TRPC1 plays a protective role. TRPC1 regulates the production of both NO and endothelin–1 (ET-1) (Figure 4) [111]. Recently, evidence strongly indicates that the loss of endothelial TRCP1 triggers aortic hypercontractility and induces hypertension [111]. Notably, the study further demonstrates that knock-in of endothelial TRPC1 can ameliorate enhanced endothelial-dependent contraction and hypertension in obese mice. TRPC1 expression levels are elevated in Ang II-treated vascular smooth muscle cells (VSMCs) [112]. TRPC1 deficiency in VSMCs attenuates Ang II-induced vasoconstriction, hypertension, and cardiac hypertrophy [113]. The study shows that Ang II-Ca^2+^ influx and activation of mitogen-activated protein kinase/extracellular signal-regulated kinase (MEK-ERK) promote VSMC proliferation and migration, contributing to hypertension and cardiovascular remodeling. Taken together, these findings highlight that aberrant TRPC1 expression and function are associated with vascular dysfunction, thus contributing to MetS.

Activation of TRPC1 by TNF–α increases vascular permeability [114]. Similarly, TRPV4 contributes to vascular permeability [115] and endothelial-dependent vasodilation [116]. TRPC3 channels are involved in both vascular smooth muscle contraction and endothelial NO release [117]. Evidence further supports the existence of TRPV4–TRPC1 heterodimeric channels that mediate the flow of shear stress-mediated calcium influx in endothelial cells [116,118].

Activation of TRPA1 by oxidative stress induces sodium and calcium influx in vascular endothelial cells, highlighting its role in ROS signaling. Moreover, TRPA1 activation at myoendothelial junctions in cerebral arteries promotes endothelial-dependent smooth muscle relaxation via activation of calcium-activated potassium channels (KCa3.1), resulting in myocyte relaxation [119]. Taken together, these studies underscore the significant involvement of TRPA1 and TRPV4 channels, particularly TRPC1, TRPC4, and TRPC5 channels, in MetS-associated pathological processes, including endothelial dysfunction, inflammation, oxidative stress, T2DM, and neurodegenerative disease.

## 5. Age-Dependent Changes in Cell Remodeling and Herbs That Influence These Changes at the Molecular Level

While MetS and aging share common risk factors, including unhealthy diet, increased central adiposity, and sarcopenia [120], both are major contributors to the development of chronic diseases such as diabetes and cardio- and cerebrovascular disorders. MetS affects approximately 40% of older adults in Ireland [121], yet nearly 60% remain unaffected. This disparity raises important questions about why certain individuals demonstrate resilience despite exposure to well-established risk factors, including central adiposity and advancing age.

Several mechanisms have been proposed to explain the high prevalence of MetS in older populations; however, these explanations have not been clearly associated with causation. Emerging evidence delineates that MetS-associated chronic subclinical inflammation accelerates epigenetic aging in older adults [122]. In parallel, adipose progenitor cells isolated from older individuals exhibit reduced lipid incorporation alongside increased oxidative stress [123,124].

The progression of aging is not uniform among individuals, either within or between ethnic communities. Aging should therefore be considered a syndrome, an array of traits rather than a singular process. Understanding the variability in traits that predispose certain individuals to a more rapid aging trajectory is central to unlocking the complexities of human aging. MetS, a constellation of interconnected physiological dysfunctions, is not universally prevalent among older adults in the Irish population, underscoring the presence of protective or resilience-associated traits that may shield against accelerated aging.

The relationship between MetS and aging is complex and non-linear, shaped by interactions among modifiable lifestyle factors, intrinsic biological processes, and genetic influences. Both are increasingly recognized as gradual and irreversible pathological processes.

**Figure 5:**
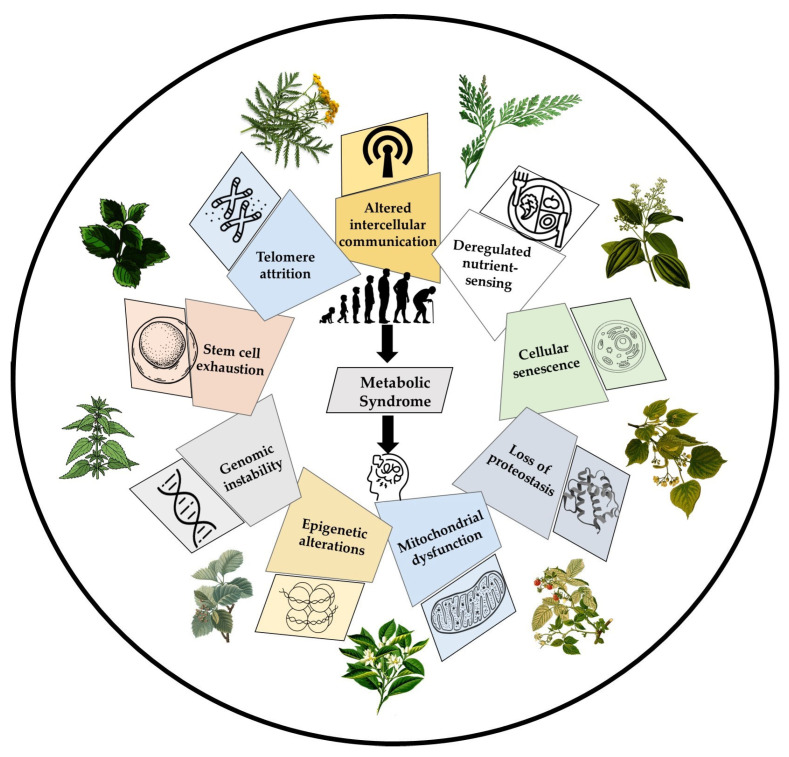
Herbal medicines influence aging at the molecular level.

Aging is delineated in the context of MetS based on the “hallmarks” of aging originally proposed by López-Otín and colleagues [125]. These hallmarks reflect harmful dysfunctions that correspond to specific aging traits. Here, we focus on nine hallmarks that describe the biological processes of aging and their important roles in the context of MetS, an aging-related disease in humans (Figure 5). This section also provides insights into the roles of bioactive compounds and herbal medicines in each hallmark of aging, which may, in turn, provide beneficial effects on cognitive decline.

### 5.1. Genomic Instability

Genomic instability refers to gradual alterations in the function and structure of DNA stemming from chemical modifications over time, which ultimately lead to impaired genome integrity [126]. Genomic instability is a fundamental process contributing to aging [127].

Genomic instability may represent a vital mechanistic bridge between MetS and age-related disease progression. In a recent systematic review, authors concluded that MetS is associated with increased DNA damage through, in particular, oxidative and nitrosative stress and impaired antioxidant defense [128]. In addition to oxidative stress-induced DNA damage in MetS, disturbances in the adenosine 5′-monophosphate-activated protein kinase (AMPK) signaling pathway may also contribute to genomic instability. AMPK plays a crucial role as a mediator linking metabolic homeostasis to DNA damage repair, particularly the base excision repair pathway via the modulation of 8-oxoguanine glycosylase [129]. Diet plays a pivotal role in genomic instability. For instance, it has been demonstrated that micronutrient deficiencies, including zinc, selenium, copper, and iron deficiencies, can lead to cognitive decline through damaging DNA [130]. In addition to trace elements, a growing body of evidence has addressed the link between natural products and herbal medicines and genomic instability.

### 5.2. Telomere Attrition

Telomeres are made up of complex structures encompassing RNA, proteins, and a tandemly repetitive DNA sequence of TTAGGG, which protects the ends of chromosomes from degradation caused by oxidative stress and cell division, as well as end-to-end fusion [131,132,133,134]. Telomere shortening is strongly linked to elevated risk of developing numerous age-associated conditions comprising CVDs [135,136], T2DM [137], and liver diseases [138]. Contrary to the generally accepted view, cross-sectional analyses have also revealed a paradoxical increase in leukocyte telomere length among patients with T2DM and MASLD [138].

Compelling research has indicated a link between telomere attrition and cognitive decline [139,140,141,142], while some studies did not find a relationship between telomere shortening and neurodegenerative disorders such as Alzheimer’s disease [143]. Furthermore, a longitudinal study on 880 subjects demonstrated that short telomeres, but not telomere attrition rates, might be a predictive indicator for aging-induced memory decline [144]. The reverse of telomere attrition can occur via the activity of telomerase, an enzyme that is active in several tissues, including lymphocytes, stem cells, and, in general, in high-proliferating cells [145,146]. Therefore, any natural or synthetic compound that exhibits telomerase activator effects may have promising anti-aging effects.

### 5.3. Epigenetic Alterations

Epigenetic is defined as “an epigenetic trait is a stably heritable phenotype resulting from changes in a chromosome without alterations in the DNA sequence” [147]. Both genetic and epigenetic alterations can lead to a wide spectrum of human diseases, such as aging, cardiovascular, and neurological diseases [148]. Epigenetic alterations include remodeling of chromatin, changes in DNA methylation patterns, histone modification, and non-coding RNAs [149]. Epigenetic alterations do not alter the DNA sequence, yet they affect gene expression and the architecture of chromosomes [150]. Epigenetic alterations are another pivotal hallmark of aging contributing to MetS-associated cognitive decline. The available evidence suggests that MetS may promote maladaptive epigenetic remodeling, which promotes systemic and possibly neurological dysfunction [151].

A growing body of literature has examined the potential impacts of natural products and plant-derived compounds on epigenetic alterations, which impact epigenetic pathways [150].

### 5.4. Loss of Proteostasis

Loss of proteostasis is one of the primary hallmarks of aging, leading to cellular aging [152]. Proteostasis or protein homeostasis refers to the quality control mechanisms by which cells maintain the functionality and stability of their proteomes [149]. The quality control mechanisms involve protein synthesis, stabilizing and accurately folding proteins, and degradation of proteins primarily by two pathways, namely the proteasome system and the autophagy–lysosomal pathways [153]. Loss of proteostasis can result in the accumulation of damaged and misfolded proteins and increased protein aggregation, overwhelming chaperone, proteasome, and autophagy systems [149,154].

In the context of MetS, loss of proteostasis is often reflected by chronic ER stress. Prolonged exposure to excess lipids, oxidative stress, and glucose can exhaust the capacity of protein-folding by the ER, resulting in a persistent activation of the unfolded protein response [155]. Loss of proteostasis can occur due to prolonged ER stress, which ultimately leads to inflammation, InsR, and tissue injury, representing an important mechanism linking MetS with accelerated aging-related decline.

Several natural products and herbal medicines are capable of restoring cellular protein homeostasis by reducing oxidative stress, suppressing chronic inflammation, and regulating pathways that contribute to the unfolded protein response.

### 5.5. Mitochondrial Dysfunction

Mitochondrial dysfunction is one of the hallmarks of aging [149], and it refers to diminished ATP generation capacity and elevated electron leakage owing to attenuated efficacy of the respiratory chain [156]. The relationship between mitochondrial dysfunction and cognitive decline can be mediated through alterations in mitochondrial morphology; disruptions in energy metabolism and oxidative stress; damage to mitochondrial DNA and RNA; disruptions in calcium homeostasis; neuroinflammation; and alterations in mitochondrial biogenesis, dynamics, and apoptosis pathways [157]. It should be noted that natural products and their active compounds that target the aforementioned pathways are capable of reversing and preventing mitochondrial dysfunction, thus hindering the progress of cognitive decline. 

### 5.6. Cellular Senescence

Cellular senescence is a biological process that entails an irreversible and permanent arrest of the cell cycle occurring in response to numerous endogenous and exogenous cellular stressors, including oxidative stress, telomere shortening, oncogene activation, and DNA damage, with the aim of halting the proliferation of damaged cells [158,159,160]. The accumulation of senescent cells due to aging can stimulate the production of pro-inflammatory proteins, also known as the senescence-associated secretory phenotype (SASP), leading to chronic inflammation, which ultimately results in metabolic diseases [161], including MetS, osteoporosis, metabolic CVD, and diabetes [162]. Cellular senescence ultimately activates cytokine-dependent kinase inhibitors, namely p16^INK4a^, p21, and p53 [158]. Therefore, natural products with the potential to attenuate and delay cellular senescence can play pivotal roles in alleviating metabolic diseases.

Although cellular senescence is one of the major drivers for metabolic diseases, there is a dearth of human studies in older adults with MetS addressing the potential role of natural products in cellular senescence.

### 5.7. Deregulated Nutrient Sensing

Nutrient-sensing pathways entail both extracellular ligands and intracellular signaling cascades, including SIRT, insulin and IGF-1, AMPK, and mTOR, all of which play a vital role in the aging process [125]. A growing body of literature has explored the association between deregulated nutrient sensing and metabolic diseases, including but not limited to MetS. For instance, recent evidence delineated a close link between dysregulation of AMPK and InsR [163,164]. It has been shown that dysregulated nutrient sensing is associated with T2DM in older adults [165,166]. Natural products targeting the aforementioned signaling pathways can mitigate deregulated nutrient sensing, resulting in substantial improvements in MetS and InsR.

### 5.8. Stem Cell Exhaustion

One of the repercussions of aging is stem cell exhaustion, leading to a substantial functional loss of tissues and diminished regenerative potential of the tissues at steady state [167]. With aging, stem cell exhaustion occurs in multiple tissues, including, but not limited to, satellite cells in muscles, intestinal epithelial stem cells, mesenchymal stem cells, and neural and hematopoietic stem cells [168]. Of particular interest, stem cell exhaustion can arise from DNA damage, telomere attrition, mitochondrial dysfunction, cellular senescence, and epigenetic alterations [149]. Ultimately, stem cell exhaustion can lead to various age-related diseases, namely muscle atrophy, neurodegeneration, immune system malfunctioning [167], frailty, osteoporosis, and diminished physical performance [169].

Recently, the potential therapeutic effects of stem cells in various metabolic disorders, including MetS, have attracted global research attention. In an animal study on high-fat diet-induced obese mice, the researchers found that mesenchymal stem/stromal cells significantly decreased body weight and improved dyslipidemia, mainly through activation of the AMPK signaling pathway in adipose tissue [170]. Other studies showed similarly promising effects of mesenchymal stem cells on glucose homeostasis, energy metabolism, and insulin sensitivity [171,172]. Furthermore, in human studies, mesenchymal stem cell therapy has been shown to improve insulin sensitivity through various mechanisms, including anti-inflammatory effects, oxidative stress, and improved β–cell functions in the pancreas [173]. In a human trial on individuals with T2DM, the combination of bone marrow mesenchymal stem cells and mononuclear cells revealed promising effects on the complications of T2DM over an 8-year follow-up, including a substantial decline in the incidence of diabetic peripheral neuropathy, as well as macrovascular complications [174]. All of these studies highlight a strong link between stem cell exhaustion and numerous components of MetS, particularly InsR.

There is a growing body of evidence linking natural products to stem cell exhaustion in stem and progenitor cell models. However, human intervention trials that directly quantify stem cell exhaustion are rare, highlighting a gap in the literature. Because stem cell exhaustion is an integrated consequence of other hallmarks of aging, including mitochondrial dysfunction, telomere attrition, DNA damage, and epigenetic alterations, natural products targeting these processes may also play a critical role in alleviating stem cell exhaustion. In this regard, several nutraceutical agents seem to converge on senescence program regulation and mitochondrial quality control. Lower doses of quercetin treatment in human exfoliated deciduous teeth stem cells exhibited promising effects on the energy metabolism of stem cells and enhanced SIRT expression [175].

Because direct human clinical trials are limited, further research is needed to address the effects of different natural products on stem cell exhaustion.

### 5.9. Altered Intercellular Communication

Aging is accompanied by altered intercellular communication, which in turn compromises the maintenance of hormesis and homeostasis [125]. Intercellular communication occurs via the release of soluble factors that influence the function of neighboring and distant cells [176]. Among intercellular communication mediators, extracellular vesicles (EVs) [177] and SASP [178] are of great importance. Exosomes are small EVs released by all cell types, playing a crucial role as important mediators of intercellular communication [179]. Given the pivotal roles of mitochondria in both intra- and intercellular communication pathways, age-related deregulation of mitochondrial communications affects all tissues [180]. Various age-related diseases are associated with altered intercellular communication, including MetS [181,182] and atherosclerosis [183]. Numerous studies have delineated that EVs can undergo significant changes in metabolic-related diseases, including obesity and MetS [184,185,186,187]. Targeting SASP has been demonstrated to reverse the complications of diabetes, including InsR and glucose homeostasis [188]. Numerous herbal medicines and natural products have been shown to positively alter intercellular communications, which in turn may help attenuate the development of MetS and InsR.

## 6. Traditional Anti-Aging Medicines with the Potential to Be Translated into Effective Treatments for MetS-Induced Cognitive Decline

**Figure 6:**
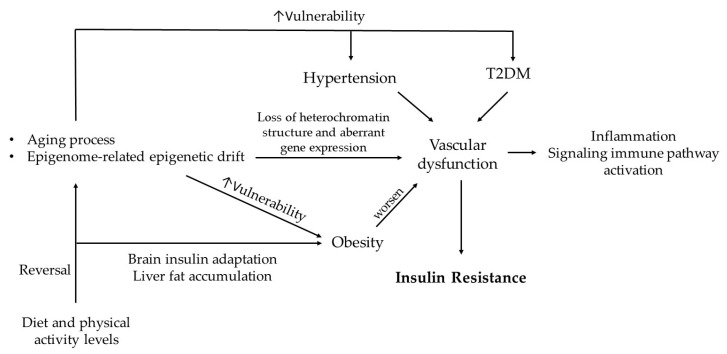
Reduced physiological and functional capacity due to aging, along with poor diet, contribute to the development of vascular dysfunction-induced cognitive decline.

The evidence strongly supports the notion that hypertension, T2DM, and obesity can induce vascular damage, reduce blood flow, and increase the risk of injury to organs such as the heart, ultimately contributing to a decline in neurovascular unit function, a process widely recognized as a key factor in age-related health decline (Figure 6). Moreover, genetic predisposition to hypertension, diabetes, and InsR may exacerbate these conditions and impair insulin-responsive target tissue function.

Phytomedicines are increasingly becoming part of evidence-based medicine and are being integrated with conventional therapies to manage these conditions. Identification of anti-aging bioactive molecules and herbal medicines with therapeutic potential for patients with MetS, while also slowing biological aging, would be valuable for preventing and managing cognitive decline.

In this section, we summarize studies published within the past five years on herbal medicines or their bioactive molecules and their anti-aging roles, identified through searches of PubMed, Scopus, and Google. The alphabetical list of herbal medicines with sound evidence suggesting potential benefits in slowing biological aging while mitigating MetS-induced cognitive decline is presented in Table 1 (preclinical) and Table 2 (clinical trials).

1. *Alpinia oxyphylla* Miq., a medicinal plant, has been reported to improve cognitive impairment in post-ischemic stroke models by inducing the BDNF (brain-derived neurotropic factor)/TrkB (tropomycin receptor kinase B)/AKT signaling pathway [190]. p-Coumaric acid (also known as 4-hydroxycinnamic acid), a bioactive compound derived from Alpinia oxyphylla Miq., promotes hippocampal neurogenesis, enhances spatial learning, and improves both short- and long-term memory. These effects are mediated through the AKT pathway and are dependent on BDNF/TrkB signaling.
2. *Astragali radix* is a widely used herb that exerts immunomodulatory [241], anti-hyperglycemic, anti-oxidant [242], anti-aging, anti-inflammatory [191], cardioprotective [243], and anti-aging effects [244], with minimum side effects [242,245]. Astragali radix contains diverse chemical constituents [246].
3. Astragaloside IV exhibits a promising capacity to attenuate mitochondrial dysfunction in podocytes through the SIRT1/PGC1α/Nrf1 pathway, and it diminishes oxidative stress [192].
4. Astragalus, a plant commonly utilized in traditional Chinese medicine, was shown to exhibit anti-aging effects through activating telomerase and inducing telomere length extension [234]. In an RCT on 40 middle-aged healthy individuals, the use of an astragalus-based supplement for 6 months significantly demonstrated longer median and shorter telomere length compared to the control group, where no changes in telomere length were exhibited [234].
5. *Atractylodis macrocephalae rhizoma* (*Atractylodes macrocephala* Koidz.), a herb approved for use as a supplement in China, has demonstrated notable neuroprotective potential in preclinical studies. Evidence indicates that its medicinal properties include gastrointestinal support, anti-aging and antioxidant effects, and promotion of blood circulation [193]. A study indicates that *Atractylodis macrocephalae rhizome* alleviates neuroinflammation in AD through enhancing the cAMP signaling pathway [194]. It counteracts cyclophosphamide-induced immunosuppression in mice and restores normal immune function [195]. Evidence also indicates that *Atractylodis macrocephalae rhizoma* may enhance lymphocyte proliferation by inhibiting the PI3K/Akt/NF-κB signaling pathway; reducing IL-6, IFN-γ, and TNF-α levels; correcting immune cell imbalances; attenuating inflammatory responses; and improving immune function in aging rats. This herb may potentially delay aging due to its ability to reduce inflammation and enhance immune function [193].
6. An 8-week RCT on 91 healthy subjects revealed the beneficial effects of Aronia melanocarpa supplementation on attenuating H_2_O_2_-induced DNA strand breaks ex vivo [235].
7. Baicalin is an active ingredient predominantly found in *Scutellaria baicalensis*, a Chinese herbal medicine, and it exhibits anticancer, antifibrotic, anti-inflammatory, antioxidant, and anti-microbial properties [199,247,248,249]. In Parkinson’s disease rat models, baicalein exhibited neuroprotective effects by ameliorating mitochondrial dysfunction and activating mitochondrial autophagy through the SIRT1/AMPK/mTOR and miR-30b-5p pathways [196]. Baicalin also delineated protective effects against heart failure via improving mitochondrial dysfunction, as evidenced by reduced ROS production, apoptosis, and cardiac fibrosis in both in vivo and in vitro models [197]. Furthermore, baicalin has been shown to inhibit hypoxia-inducible factor-1α, thus reversing mitochondrial dysfunction and improving aerobic glycolysis [198]. It has been shown that baicalin is capable of preventing atherosclerosis via regulating the SIRT1/NF-κB signaling pathway mediated by exosomes stemming from the treatment of mesenchymal stem cells by baicalin [199].
8. Bazi Bushen, a traditional Chinese medicine, has been shown to activate SASP, leading to significant alterations that impede inflammation-related pathways, including arachidonic linoleic acid metabolism and TNF- and IL-17-induced inflammatory pathways [200].
9. *Centella asiatica* (L.) Urban, also known as gotu kola, a herb belonging to the Apiaceae family and widely grown in tropical and subtropical regions [205,250,251], has also been shown to have anti-aging effects via upregulating the activity of telomerase in human peripheral blood mononuclear cells (PBMCs) [252].
10. *Curcuma longa* plant. It should be noted that not all natural products have promising effects on telomere maintenance. One of these natural products is curcumin, which is a polyphenolic compound derived from the Curcuma longa plant [253]. Curcumin, found primarily in the turmeric root [254], has been found to promote telomere attrition via inhibiting telomerase activity in tumor cells [255]. In addition, epigallocatechin gallate (EGCG), a catechin derived from green tea [256], promotes telomere shortening via genotoxicity [257]. Curcumin has been shown to affect DNA methylation by inhibiting DNA methyltransferase 1 (DNMT1) to modulate histone modification via inhibition of histone acetyltransferases [150]. On the other hand, in a study conducted in human leukemia cells, the authors found that curcumin and its derivatives can alter histone methylation and the activity of histone methylation/demethylation enzymes, and this is highly dependent on context and cell types [202].

Several studies have examined the effects of EGCG on epigenetic alterations. It has been shown that EGCG can suppress DNMT, ultimately leading to an alteration in the DNA methylation status [258]. In addition, EGCG can affect epigenetic alterations by elevating histone 3 and 4 and decreasing histone deacetylase (HDAC) activity via modulating the gene expression of HDAC2, histone methyltransferase (G9a), and HDAC1 [259]. Emodin, an anthraquinone primarily found in knotweed, rhubarb, buckthorn, and Da Huang, can also target HDAC activity, at least in part, through chelating zinc ions within HDAC catalytic domains, resulting in histone acetylation [260]:

11. Mylife/Mylife100^®^ dietary supplement. In an 8-week RCT on 32 middle-aged Thai adults, Mylife/Mylife100^®^ dietary supplement significantly increased the average telomere length between the baseline and the 8-week time point, possibly through the antioxidant properties of its ingredients [236]. This dietary supplement consists of soy protein, guava fruit, mangosteen aril, black sesame seed, and pennywort leaves. In another study, fortified mangosteen extract exhibited anti-aging properties via slowing telomere shortening [261].
12. Flavonoids, common constituents of fruits and Chinese herbal medicines, have been shown to protect vascular homeostasis through their antioxidant, anti-inflammatory, and antiaging effects in both in vitro and in vivo studies [250]. One study demonstrated that luteolin, a flavonoid, activates eNOS and increases NO production, resulting in concentration-dependent relaxation of vascular tension in rat aortic rings. Another study reported that luteolin-7-O-glucoside exerts antiproliferative and significant antioxidant effects by inhibiting signal transduction and the activator of the transcription 3 (STAT3) pathway [205].
13. Fangji Huangqi Decoction, a traditional Chinese medicine, demonstrated a significant elevation in mitophagy in podocytes in rat models via increasing BNIP3 expression [203].
14. Fisetin is a naturally occurring flavone with senolytic activity via upregulating anti-apoptotic pathways [205]. Fisetin has been shown to target various canonical indicators of cellular senescence in adipose-derived stem cells, including ROS, senescence-associated β–galactosidase, and senescence-associated heterochromatin foci [204].
15. Ginseng (*Panax ginseng* C.A. Meyer), a long-lived perennial herb belonging to the family of Panax (Araliaceae), is one of the most commonly used herbal nutritional products, known to promote vitality and longevity, reduce stress, fatigue, and weakness, and support both mental and physical health [251]. Recent evidence demonstrated that ginseng callus cells subcultured for 12 consecutive years retained chromosomal stability and totipotency, with minimal decline over time [252].
16. Grape Seed Extract: In contrast to Bazi Bushen, procyanidin C1, a polyphenolic component of grape seed extract, can inhibit SASP at lower concentrations and selectively eliminate senescent cells at higher concentrations [178].
17. *Jiang Gui Fang* increases the core temperature of mice by activating interscapular brown adipose tissue (iBAT) and inducing the browning of epididymal WAT (eWAT). This effect appears to be mediated through the peroxisome proliferator-activated receptor gamma (PPARγ)/SIRT1–PPARγ coactivator-1α (PGC-1α) signaling pathway [206].
18. Jingfang Granule, a traditional Chinese medicine, is often used for the treatment of infectious diseases. *Jingfang Granule* is a blend of 11 herbs. A recent study shows that Jingfang Granule not only significantly increases the median lifespan of C. elegans by 31.2% at a dosage of 10 mg/mL but also enhances oxidative stress resistance by reducing ROS levels [209]. Jingfang Granule also delays reproductive senescence in C. elegans. The authors propose that Jingfang Granule protects C. elegans from oxidative stress, thereby extending its lifespan. *Jingfang Granule* effectively promotes wound healing in diabetic rats [208]. It exerts anti-inflammatory and proangiogenic effects in vitro. American ginseng (*Panax quinquefolius*) maintained proteostasis through enhancing the activity of cathepsin B, a lysosomal protease in the autophagy–lysosomal pathway, leading to the removal of damaged proteins, as well as enhanced autophagic flux [262].
19. *Lycium barbarum* L. (*L. barbarum*), commonly known as wolfberry or goji berry, is widely used in traditional Chinese medicine. Among the multitude of its therapeutic effects, various strains of *L. barbarum* contain bioactive compounds that target 90 aging-related genes, providing evidence that they may represent a molecular source for anti-aging and age-delaying properties [193]. Evidence indicates that this herb possesses antioxidant, immunomodulatory, and glycemic-regulating properties, making it a promising candidate for addressing metabolic and obesity-related health challenges [263].
20. Scarlet beebalm (*Monarda didyma* L.), a perennial plant predominantly grown in Canada and the US and belonging to the Lamiaceae family, is rich in essential oil and phenolic compounds [264]. *M. didyma* L. contains didymin, a flavonoid glycoside that contributes to its anti-aging, antioxidant, and anti-inflammatory properties [265]. An RCT found that daily supplementation with M. didyma meaningfully stabilized DNA methylation age and improved telomere length [213].
21. *Monochoria angustifolia*, or Siam violet pearl, is the newest species of the genus Monochoria C. Presl, found in Thailand, and has long been consumed as food and in herbal medicine. It has been shown to produce apigenin-7-O-glucoside, an abundant antioxidant phytochemical [215], and may also have anti-inflammatory effects. Recent in silico and in vitro studies indicate that apigenin-7-O-glucoside is a potential anti-aging agent due to its ability to inhibit collagenase and elastase [216]. Since these enzymes contribute to tissue damage in diabetes, their inhibition by apigenin-7-O-glucoside may help reduce tissue damage in diabetic patients. The authors also provide evidence suggesting that the compound is safe based on its pharmacokinetic properties. However, further studies are needed to fully understand its therapeutic potential in the management of MetS.
22. *Nicandra physalodes* extract prolongs both lifespan and health span in C. elegans and ameliorates cellular senescence in human fetal lung fibroblasts (MRC-5 cells) [217]. It also counteracts premature aging in doxorubicin-treated aging mice. Treatment with *Nicandra physalodes* extract reverses liver function damage and reduces senescence marker levels, including ALT, SA-β-Gal, and γH2AX. The protective and antioxidative effects of this herb are mediated through insulin signaling pathways, involving DAF-16 and HSF-1. Naringenin, a flavanone predominantly found in citrus fruits, has been shown to exhibit promising effects on epigenetic alterations [266].
23. Nicotinamide Riboside: In a study on older adults with mild cognitive impairment, nicotinamide riboside, a vitamin B3 derivative, significantly increased blood levels of NAD+, indicating the potential role of this product in nutrient-sensing networks [237].
24. *p-Coumaric acid* is a 4-hydroxycinnamic acid derivative that is abundant in Chinese herbal medicines. It plays a role in oxidative stress-related diseases [267], including inflammation [218], CVDs [268], diabetes, MASLD [269], and nervous system disorders, according to recent reviews [244,270]. One study further indicates that the combination of metformin and p-coumaric acid improves MASLD by decreasing lipid accumulation and inhibiting inflammation [271]. p-Coumaric acid also increases outer and plasma membrane permeability in bacteria [272], which may potentially affect endothelial structure and function.
25. Pomegranate Extract: In a 12-week RCT on older adults, pomegranate extracts demonstrated beneficial effects on circulating levels of IGF-1, with no changes in telomere length [238]. IGF-1 has been shown to have potentially protective effects on vascular aging by mitigating oxidative stress and inhibiting signaling pathways associated with inflammation and apoptosis [273,274,275].
26. Quercetin: An RCT of quercetin, a natural flavonoid, in male patients with coronary artery disease found a significant decrease in vascular senescence and inflammaging signatures, indicating potential sex-specific effects of quercetin on regulating senescence-associated biology in humans [239].
27. Resveratrol, a polyphenol found primarily in certain berries, grains, roots, seeds, tea [276], Japanese knotweed, grapes, and red wine, has been shown to demonstrate anti-aging effects via telomere maintenance [254]. Resveratrol is capable of upregulating telomerase reverse transcriptase (hTERT) and stimulating silent information regulator proteins (SIRs) and their homologs, known as sirtuin (SIRT), along with the Nrf2 (nuclear factor erythroid 2-related factor 2) signaling pathway in human HepG2 hepatocellular carcinoma cells [220].

Resveratrol can inhibit the activity of HDAC and histone acetyltransferase [260]. It has also been shown to enhance energy production, trigger mitochondrial biogenesis, and stabilize mitochondrial fission–fusion dynamics via activation of the SIRT1/SIRT3-Foxo pathway [221]. In an RCT, resveratrol has been found to significantly increase circulating levels of SIRT1 in older adults with T2DM [240]:

28. Salidroside, a phenylpropanoid glycoside derived from the plant *Rhodiola rosea* L., is capable of increasing the expression of SIRT1 and inducing autophagy via the AMPK-SIRT1 pathway [222] and mTOR [223]. It also reduces the expression levels of IGF-1 and regulates the insulin/IGF-1 signaling pathway [224]. Thus, salidroside may mitigate MetS and improve InsR via attenuating deregulated nutrient sensing. Moreover, salidroside has been shown to exert protective effects against liver fibrosis via upregulating miR-146a-5p, a major component of human liver stem cell exosomes [277], which ultimately inhibits hepatic stellate cell activation [278].
29. *Si Jun Zi Tang* (SJZT) is composed of four herbal medicines: Ginseng Radix et Rhizoma, *Atractylodis macrocephalae Rhizoma*, Poria, and Glycyrrhizae Radix et Rhizoma. SJZT is a classic traditional Chinese medicine prescription used to treat aging-related diseases, including skin disease. One study identified 131 bioactive compounds that met absorption, distribution, metabolism, and excretion (ADME) parameters, along with 235 target genes associated with aging [226]. According to the Kyoto Encyclopedia of Genes and Genomes (KEGG), the anti-aging mechanism of SJZT appears to be mediated through inhibition of the PI3K-AKT and p38 MAPK signaling pathways [226]. Thus, SJZT is a potential anti-aging herbal medicine. Sulforaphane, an isothiocyanate abundantly found in broccoli and other cruciferous vegetables, has been shown to affect DNA methylation [273].

The PI3K-AKT pathway plays a key role in clearing glucose from the blood and restoring glucose homeostasis. A study has shown that the inhibition of PI3K can cause hyperglycemia and an increase in fasting C-peptide [279]. While PI3K α and PI3K β isoforms are involved in glucose uptake in muscles, they also mediate gluconeogenesis in the liver [280]. In contrast, PI3K γ isoform responses are not only involved in glucose metabolism [280] but also regulate the migration, differentiation, and activation of myeloid-lineage immune cells, highlighting the diverse physiological roles of PI3K isoforms [281]. Although the PI3K γ isoform is highly expressed in leukocytes [282], it is also present in muscle tissue [280]. PI3Kγ-deficient macrophages and monocytes exhibit increased production of inflammatory mediators [282].

SJZT lacks PI3K subtype selectivity, which may lead to side effects based on our review of the literature. Inhibition of the PI3K pathway by SJZT may therefore exacerbate or prolong hyperglycemia and inflammation in patients with MetS. Further characterization of SJZT and the development of PI3K isoform selectivity in insulin-responsive target tissues are necessary to demonstrate its therapeutic efficacy in patients with MetS.

Another study indicated that SJZT reduces ROS generation and oxidative stress, increases mitochondrial membrane potential, and upregulates the expression of stem cell markers in vitro [227]. SJZT was also found to suppress the expression of p53, p-p53, and p21 and downregulate p38 phosphorylation. Importantly, the anti-cellular senescence effect of SJZT in eliminating epidermal stem cells (EpiSCs) harboring genomic lesions disappeared after treatment with the p38 inhibitor adesmapimod. This study also identified key active components, glycyrrhizin, ginsenoside Rg5, ginsenoside Rh2, liquiritin, polyporenic acid C, and atractylenolide II, which exhibited strong affinity for key proteins involved in cellular senescence signaling. Interestingly, SJZT also modulates intestinal flora composition, contributing to its immunomodulatory effects [225]:

30. Spermine and spermidine, which are polyamines, have been shown to have neuroprotective effects via stimulating autophagy [283], which subsequently results in reducing the aggregation of Tau and αS in neurons and microglia [284]. A cross-sectional study of 2674 older adults demonstrated a significant association between dietary intake of spermine and improved cognitive function [285]. Spermidine has been shown to effectively protect mesenchymal stromal cells against oxidative stress and exhibit antisenescence effects, at least in part, through SIRT3 [228].
31. Syringin, a phenylpropanoid glucoside found predominantly in the medicinal plant Acanthopanax senticosus, exerts antioxidant and anti-inflammatory activities [229]. Syringin has been demonstrated to stimulate autophagy, at least in part, by modulating the miR-34a/SIRT1/Beclin-1 axis, and to inhibit apoptosis induced by 6-hydroxydopamine in Caenorhabditis elegans models [230]. SIRT1 plays a crucial role in mitophagy and mitochondrial biogenesis [286,287].
32. Theaflavins, functional phytochemicals found primarily in black and dark tea, have beneficial effects on MetS [288]. Theaflavins act on multiple signaling pathways targeting dyslipidemia, obesity, and hyperglycemia. For instance, theaflavin TF3 activates the AMPK signaling pathway, leading to a reduction in hepatocyte lipid deposition [231].
33. Thymoquinone is an active ingredient primarily found in Nigella sativa seeds [289], and it exhibits protective effects against neuro-related disorders by ameliorating Aβ-induced neurotoxicity and mitochondrial membrane depolarization. These effects are mediated through inhibition of ROS formation and reduction in oxidative stress, as well as prevention of apoptosis via modulation of mitochondrial function and a decrease in levels of cytochrome-C and caspase-3 [289]. Similarly, in a study on rats, thymoquinone ameliorated inflammation, apoptosis, and oxidative stress, and it preserved mitochondrial DNA contents in cardiomyocytes [232].
34. *Wuzi Yanzong* Pill (WYP) is a traditional herbal prescription widely used in the treatment of male infertility. It consists of several herbs, including Gouqizi (Fructus Lycii), Tusizi (Semen Cuscutae), Wuweizi (Fructus Schisandrae Chinensis), Fupenzi (Fructus Rubi Chingii), and Cheqianzi (Semen Plantaginis). WYP exhibits neuroprotective and anti-inflammatory properties [290]. Although its precise molecular mechanisms remain unclear, evidence suggests that WYP exerts protective effects on nerve cells. Studies indicate that WYP appears to inhibit apoptosis and enhance the secretion of neurotrophic factors through activation of the PI3K/AKT signaling pathway [233].

In addition, clinical studies have demonstrated that WYP can decrease elevated semen DNA fragmentation index (DFI) and ROS levels, increase SOD activity, and improve sperm DNA integrity [291]. The study also indicates that WYP inhibits liver injury, lowers blood sugar and blood lipid levels, and appears to exert anti-aging and immunomodulatory effects.

In summary, these herbs can be categorized as “biologically based practices” and may be regulated as drugs, dietary supplements, or foods according to the National Center for Complementary and Alternative Medicine (NCCAM) guidelines [292]. Multiple herbal medicine options for antiaging treatment are increasingly becoming available in various countries, but there is often a lack of evidence from head-to-head preclinical studies to determine whether one herb is more efficacious than another or than other existing reported herbs. Moreover, robust and well-designed clinical studies are required to support the integration of many of these herbal medicines into standard treatment regimens for the antiaging indications reviewed here. Nonetheless, their long-standing use among diverse ethnic populations may help pave the way for future therapeutic applications and support their potential role as drugs or dietary supplements.

## 7. Conclusions

Over the past three decades of research in the glucose metabolism field, it has become clear that insulin and its signaling machinery play numerous critical physiological and pathophysiological roles in MetS. Mutations in the IR gene reduce receptor number or function, leading to hyperinsulinemia, marked glycemic instability (alternating hypoglycemia and hyperglycemia), and severe InsR, as observed in individuals with Donohue syndrome. Clinically, this syndrome is characterized by multiple abnormalities, most notably a paucity of subcutaneous adipose tissue and marked muscle atrophy.

**Figure 7:**
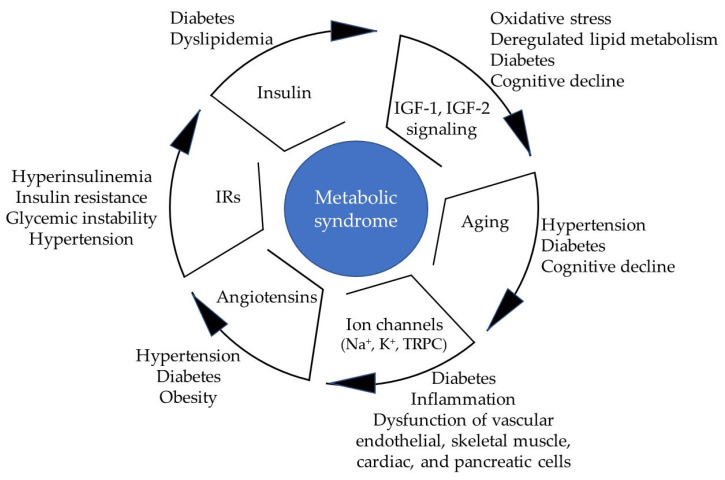
Risk factors contributing to the development and progression of cognitive decline associated with MetS. Insulin and IGF-1 signaling are closely interrelated, and modulation of vascular IGF-1 signaling can lessen the adverse vascular effects of InsR. Reduced IGF-2 receptor expression, a critical regulator of insulin secretion, cell proliferation, and autophagy, has been reported in islets from individuals with T2DM. Angiotensin-dependent AT1 and AT2 receptors also interact with insulin-signaling pathways, regulating insulin’s microvascular and metabolic actions in muscle. Clinical observations indicate that the balance between AT1R and AT2R activity is markedly altered in hypertension, obesity, and diabetes, the three major components of MetS. Disruption of ion channel function impairs the physiology of cardiac, vascular endothelial, skeletal muscle, and pancreatic cells, leading to defective glucose-induced insulin secretion. Consequently, structural and functional alterations in any key regulator of electrolyte and fluid balance, glucose metabolism, or insulin secretion can compromise the normal function of the others.

The image shown in Figure 7 illustrates the interactions among the insulin secretory machinery, IRs, insulin, IGFs, aging, ion channels, and angiotensin-related signaling pathways, which are discussed in this review, along with their potential roles in MetS-induced cognitive decline.

Structural and functional abnormalities in ion channels can disrupt the physiology of the heart and pancreas, as well as vascular endothelial and smooth muscle cells. Such disturbances may impair cardiac performance, promote inflammation, alter vasomotor function, and contribute to abnormal cellular growth. Collectively, these abnormalities in turn can intensify vascular risk and accelerate tissue and organ dysfunction in individuals with MetS.

The use of pharmacological tools has been instrumental in slowing or preventing the progression of MetS components. However, factors such as drug specificity, potential ion channel gene polymorphisms, and alterations in the expression of genes involved in glucose and lipid metabolism, as well as the RAS, must be carefully considered on an individual basis. In recent years, advances in the identification of new therapeutics, along with modifiable extrinsic factors, have significantly improved the management of hypertension, diabetes, and dyslipidemia.

Here, we discuss that *Si Jun Zi Tang*, salidroside, and resveratrol influence several conserved longevity pathways, including AMPK, the insulin/IGF-1 signaling pathway, mTOR, FoxO, and sirtuins. *Nicandra physalodes* has been shown to enhance stress resistance and delay the progression of neurodegenerative diseases through DAF-16-mediated mechanisms. Syringin, a bioactive compound derived from *Acanthopanax senticosus*, appears to promote neuroprotective effects by modulating the miR-34a/*SIRT1*/Beclin-1 axis and inhibiting apoptosis induced by 6-hydroxydopamine. In addition, *Jiang Gui Fang* has been reported to promote adipose tissue browning, which is associated with improved glucose metabolism, enhanced lipid metabolism, and reduced lipid droplet accumulation. The development of antiaging bioactive compounds and herbal medicines has emerged as a promising direction for therapeutic strategies aimed at mitigating the effects of aging and managing MetS, which is associated with increased risk for developing dementia; however, this field still requires further development.

Despite tremendous progress over the last two decades, our understanding of the mechanisms by which traditional herbal medicines influence human health and disease remains incomplete. Thus, while we celebrate the discovery of potent antihypertensive, antidiabetic, anti-inflammatory, and lipid-lowering drugs, as well as traditional medicines, new research is still needed to address the myriad unanswered questions that remain. Although clinical studies on MetS still require a lot of effort, the field remains highly promising for future therapeutic development. Nonetheless, the use of traditional herbal medicine to modulate these risk factors and enhance tissue resilience against cytotoxic stress may offer the potential for mitigating cognitive decline.

## Figures and Tables

**Table 1:** Summary of experimental studies on bioactive molecules and herbal medicines that offer therapeutic benefits in managing MetS, promoting anti-aging effects, and improving clinical outcomes.

Herb/Bioactive	Main Bioactive Compound	Study Subjects	Experimental Setting/Model	Study Design	Mechanism of Action	Pharmacological Effects	Ref.
Alpinia oxyphylla Miq	Diverse chemical constituents	Not applicable (in silico study)	Preclinical–in silico	Compound–target–pathway–disease/protein–protein interaction network constructions	Regulating the synthesis, release and transmission of neurotransmitters	Ameliorated mild cognitive impairment	[189]
		Neural stem cells; MCAOrats	Preclinical–mixed in vitro/in vivo	In vitro neural stem cell (NSC) experiments and post-middle cerebral artery occlusion ischemic rats	Activated BDNF/TrkB/AKT signaling pathway, promoted NSC proliferation	Promoted hippocampal neurogenesis, improved cognitive functions	[190]
Astragali radix	Diverse chemical constituents	LPS-stimulated IPEC-J2 cells; BALB/c mice	Preclinical–mixed in vitro/in vivo	In vitro and in vivo experimental study	Anti-inflammatory effects via inhibition of NF-κB and MAPK signaling	Reduced IL-6, IL-1β, and TNF-α; improved jejunal morphology	[191]
Astragaloside IV		db/db mice; phenyl sulfate-treated podocytes	Preclinical–mixed in vitro/in vivo	In vivo and in vitro experimental study	Attenuated oxidative stress and mitochondrial dysfunction via activation of the SIRT1/PGC1α/Nrf1 signaling pathway	Reduced proteinuria and kidney damage, lowered ROS, increased antioxidant enzymes, and improved mitochondrial biogenesis/function	[192]
*Atractylodis Rhizoma*	Diverse chemical constituents	Naturally aging rats	Preclinical–in vivo	In vivo experimental study	Anti-immunosenescence and immunomodulatory effects via inhibition of the PI3K/Akt/NF-κB signaling pathway	Improved lymphocyte proliferation, cytokine balance, immune function, and aging-related indicators	[193]
		AD rats; HT22 cells	Preclinical–mixed in vitro/in vivo	In vivo animal study with in vitro validation	Activation of cAMP/CREB/BDNF signaling and inhibition of neuroinflammation	Improved cognition, reduced neuronal damage, lower IL-6, IL-1β, and TNF-α	[194]
		BALB/c female mice	Preclinical–in vivo	In vivo experimental animal study	Upregulation of CD28/IP3R/PLCγ-1/AP-1/NFAT signaling; immunomodulatory effects	Alleviated cyclophosphamide-induced immunosuppression, improved spleen index, reduced splenocyte damage, and restored cytokine balance	[195]
Baicalein		6-OHDA-induced Parkinson’s disease rats	Preclinical–in vivo	In vivo experimental animal study	Activated mitochondrial autophagy via miR-30b-5p and the SIRT1/AMPK/mTOR pathway	Improved neuronal injury, restored dopamine-related changes, reduced apoptosis, and alleviated mitochondrial dysfunction	[196]
		ISO-induced heart failure BALB/c mice; HL-1 cardiomyocytes	Preclinical–mixed in vitro/in vivo	In vivo and in vitro experimental study	Improved mitochondrial fusion/fission balance and inhibited the GRP78/CHOP pathway, thereby reducing oxidative stress and apoptosis	Reduced ROS, apoptosis, and cardiac fibrosis; improved cardiac function in heart failure	[197]
		Tamoxifen-resistant breast cancer cells	Preclinical–in vitro	In vitro experimental cell study	Inhibited HIF-1α-mediated aerobic glycolysis and reversed mitochondrial dysfunction	Resensitized resistant cells to tamoxifen and reduced stem cell-like characteristics	[198]
		High-fat-diet-induced atherosclerosis model; ox-LDL-induced VSMCs; mesenchymal stem cell-derived exosomes	Preclinical–mixed in vitro/in vivo	In vivo and in vitro experimental study	Exosome-mediated anti-atherosclerotic effects via regulation of the SIRT1/NF-κB signaling pathway	Reduced plaque formation/progression and suppressed inflammatory responses associated with atherosclerosis	[199]
*Bazi Bushen*	Multi-component traditional Chinese medicine formula	Naturally aged mice	Preclinical–in vivo	In vivo experimental animal study	Improved age-related energy metabolism by modulating SASP-associated IL-17/TNF inflammatory pathways and arachidonic acid–linoleic acid metabolism	Reduced inflammation in metabolic organs and improved metabolic homeostasis in aged mice	[200]
*Centella asiatica*	Diverse chemical constituents	Mouse models of aging	Preclinical–in vivo	In vivo experimental animal study	Antioxidant regulatory transcription factor NRF2	Enhanced plasticity and improved cognitive function	[201]
*Curcumin analog*	Dimethoxycurcumin (DMC); curcumin comparator	HL60, U937, and Kasumi-1 leukemia cells	Preclinical–in vitro	In vitro experimental cell study	Inhibited histone lysine methyltransferases targeting H3K4, H3K9, and H3K27 and increased histone lysine demethylase activity (LSD1, JARID1, JMJD2), thereby modulating histone methylation marks	Altered histone methylation/acetylation landscape and supported epigenetic anticancer activity in leukemia cells	[202]
*Fangji Huangqi* decoction		Podocytes in rat models	Preclinical–mixed in vitro/in vivo	In vitro experimental cell study	Elevation in mitophagy, increased BNIP3 expression	Increased degradation of damaged mitochondria may provide potential therapeutic strategy for aging-related conditions	[203]
Fisetin	Flavone with senolytic activity		Preclinical–in vitro	In vitro experimental cell study	Upregulated anti-apoptotic pathways	May provide potential therapeutic strategies to reduce vascular senescence and inflammation	[204]
Flavonoids	Diverse chemical constituents	HUVEC cells	Preclinical–in vitro	In vitro experimental cell study	Anti-inflammatory and antioxidant effects via inhibition of STAT3 signaling and reduced ROS generation	Reduced STAT3 activation, proliferation, ROS, and inflammatory/oxysterol-related mediators	[205]
Grape seed extract	Procyanidin C1	Senescent human stromal cells; treatment-damaged tumor microenvironment; aged mice	Preclinical–mixed in vitro/in vivo	In vitro and in vivo experimental study	Dose-dependent senotherapeutic activity: inhibited SASP at low concentrations and selectively eliminated senescent cells at higher concentrations, partly through ROS and mitochondrial dysfunction	Reduced senescent cell burden, improved physical function, and increased lifespan in mice	[178]
*Jiang Gui Fang*	Diverse compounds	Mice, tissues	Preclinical–in vivo	In vitro and in vivo experimental study	The increase in core temperature through the activation of iBAT and the browning of eWAT appears to be mediated by the PPARγ/SIRT1–PGC-1α signaling pathway	Protected liver, and reduced glucose and lipids, reduced the content of lipid droplets and ATP in brown fat cells, elevated PPARγ and lipolytic protein hormone-sensitive triglyceride lipase	[206]
Jingfang granule	Diverse compounds	Adult Caenorhabditis elegans	Preclinical–in vivo	In vivo experimental study	Increases extracellular transcription of extracellular matrix, stress-activated transcription factor 1	Extend lifespan, slows down the functional degradation of mitochondria, enhances innate immunity	[207]
		Streptozotocin-induced diabetic rats; HaCaT cells; HUVECs	Preclinical–mixed in vitro/in vivo	In vivo and in vitro experimental study with network pharmacology validation	Anti-oxidative, anti-inflammatory, and pro-angiogenic effects via PI3K-AKT and MAPK signaling	Promoted wound healing, improved metabolic parameters, reduced inflammation/oxidative stress, and increased angiogenesis	[208]
		Caenorhabditis elegans N2	Preclinical–in vivo	In vivo experimental animal study	Anti-aging and anti-infective effects associated with reduced oxidative stress and improved stress resistance	Extended lifespan, lowered ROS, and improved survival after bacterial infection	[209]
*Lycium barbarum* L.	Diverse compounds	Rat, aortic endothelial cells	Preclinical–in vivo	In vitro experimental study	SIRT3/CypD pathway	Protect mitochondrial function	[210]
	Lycium barbarum polysaccharides	Rat aortic endothelial cells	Preclinical–in vitro	In vitro experimental study	Anti-oxidative and anti-apoptotic effects via increased SOD and NO, reduced MDA, upregulation of Bcl-2, and downregulation of Bax	Endothelial protection against oxidative injury	[211]
	Diverse compounds	Borderline hypertensive rats	Preclinical–in vivo	In vivo experimental animal study	Downregulation of renal endothelial lncRNA sONE and increased eNOS expression	Reduced blood pressure in salt-sensitive hypertensive rats	[212]
*Monarda didyma* L. *extract*	*Monarda didyma* L. extract (botanical extract; specific single active compound not isolated)	Human fibroblasts, keratinocytes, HUVECs, dermal microvascular endothelial cells; middle-aged adults in intervention group (n = 40) and placebo group (n = 41)	Preclinical–in vitro plus clinical trial	In vitro studies plus RCT	Antioxidant, DNA-protective, anti-senescence, and endothelial-protective effects; reduced telomere shortening and supported maintenance of DNAmAge	Reduced DNA damage and cellular senescence in vitro; improved endothelial function; increased leukocyte telomere length, stabilized DNAmAge, and improved quality of life in the clinical trial	[213]
*Monochoria angustifolia*	Diverse compounds	Monsonia angustifolia plant samples from Gauteng and Limpopo provinces	Preclinical–in vitro	Phytochemical/metabolomic profiling study with in vitro antioxidant assays	Antioxidant activity and presence of metabolites with potential anti-amyloid-beta relevance	Increased phytochemical content and antioxidant activity under higher abiotic stress conditions	[214]
		Plant samples from 25 natural populations in Thailand	Preclinical–in vitro	Phytochemical profiling study with in vitro and cellular antioxidant assays	Antioxidant activity mainly via hydrogen atom transfer; combined action of multiple flavonoids/phytochemicals	Higher flavonoid content and antioxidant potential	[215]
		Plant-derived flavone compound; non-human enzyme assays	Preclinical–in silico/in vitro	In silico and in vitro experimental study	Anti-aging activity through inhibition of collagenase and elastase, with weaker tyrosinase inhibition	Anti-collagenase and anti-elastase effects; potential anti-aging phytochemical	[216]
*Nicandra physalodes*	Extract	Caenorhabditis elegans Human fibroblasts (MRC-5 cells), mouse	Preclinical–mixed in vitro/in vivo	In vivo and in vitro experimental study	Longevity effect mediated through DAF-16 (regulates oxidative stress tolerance, an insulin signaling pathway) and HSF-1 (an insulin signaling pathway) Reversed liver function damage and reduced senescence marker levels (ALT, SA-β-Gal, and γH2AX)	Improved health span, enhanced stress resistance, and delayed the progression of neurodegenerative diseases. Ameliorated senescence	[217]
P-coumaric acid		Rats; adjuvant-induced arthritic rat model	Preclinical–in vivo	In vivo experimental animal study	Anti-inflammatory and immunomodulatory effects via reduction of TNF-α and circulating immune complexes	Decreased synovial TNF-α, reduced immune complexes, and modulated immune responses	[218]
		High-fat-diet-fed C57BL/6J mice; NCI-H716 and STC-1 intestinal L cells	Preclinical–mixed in vitro/in vivo	In vivo and in vitro experimental study	Promoted GLP-1 secretion via the NPM1/GRP78/Ca^2+^ signaling axis	Improved glucose–lipid metabolism, reduced weight gain, and alleviated NAFLD	[219]
Resveratrol		HepG2 human hepatocellular carcinoma cells	Preclinical–in vitro	In vitro experimental cell study	Upregulated hTERT expression, possibly via SIRT1/Nrf2 signaling	Increased hTERT expression and antioxidant signaling	[220]
		Myocardial ischemia/reperfusion injury model; H/R-induced neonatal rat cardiomyocytes	Preclinical–mixed in vitro/in vivo	In vivo and in vitro experimental study	Reestablished mitochondrial quality control via the Sirt1/Sirt3-Mfn2-Parkin-PGC-1α pathway, with effects on mitochondrial dynamics, mitophagy, bioenergetics, and oxidative stress	Reduced myocardial ischemia/reperfusion injury, improved mitochondrial function and mitophagy, and decreased oxidative damage	[221]
Salidroside		C57BL/6 mice with APAP-induced liver injury; APAP-treated L02 hepatocytes	Preclinical–mixed in vitro/in vivo	In vivo and in vitro experimental study	Activated Sirt1/Akt/Nrf2 signaling and suppressed the NF-κB/NLRP3 inflammasome axis	Alleviated acetaminophen-induced hepatotoxicity, reduced oxidative stress and apoptosis, and improved liver injury markers	[222]
		AGS human gastric cancer cells; gastric cancer xenograft model	Preclinical–mixed in vitro/in vivo	In vitro and in vivo experimental study	Induced apoptosis and protective autophagy through inhibition of the PI3K/Akt/mTOR pathway	Inhibited gastric cancer cell growth, promoted apoptosis, and triggered protective autophagy	[223]
		*Nothobranchius guentheri* (annual fish)	Preclinical–in vivo	In vivo experimental animal study	Modulated the insulin/IGF-1 signaling pathway in late-onset dietary intervention	Improved aging-related parameters and supported anti-aging effects in the annual fish model	[224]
Si Jun Zi Tang	Diverse compounds	Human fecal microbiota samples	Preclinical–in vitro	In vitro experimental fermentation study	Immunomodulatory effects via modulation of gut microbiota and short-chain fatty acid production	Altered intestinal bacterial composition and increased acetic acid/total SCFAs after incubation	[225]
		Naturally aging mice	Preclinical network pharmacology plus in vivo	Network pharmacology study with in vivo validation	Anti-aging effects via inhibition of PI3K-AKT and P38 MAPK signaling pathways	Improved aging-related signs, including osteoporosis and hair loss	[226]
		UVB-induced skin-aging model	Preclinical network pharmacology plus in vivo	Network pharmacology study with experimental validation	Anti-skin-aging effects via p38/p53 signaling	Reduced UVB-induced skin-aging changes; potential cosmeceutical application	[227]
Spermidine		Multipotent mesenchymal stromal cells; aging mouse model	Preclinical–mixed in vitro/in vivo	In vitro and in vivo experimental study	Delayed cellular senescence through SIRT3-mediated antioxidation	Reduced senescence, decreased oxidative stress, and improved stem-cell function	[228]
Syringin		Rat myocardial ischemia/reperfusion model; H9c2 cardiomyocytes	Preclinical–mixed in vitro/in vivo	In vivo and in vitro experimental study	Anti-inflammatory and antioxidant effects via regulation of SIRT1 signaling and activation of the NRF2/HO-1 pathway	Improved cardiac function, reduced infarct size and cardiac injury, decreased inflammatory cytokines and ROS, and increased antioxidant enzyme expression	[229]
		SH-SY5Y cells; Caenorhabditis elegans Parkinson’s disease model	Preclinical–mixed in vitro/in vivo	In vitro and in vivo experimental study	Protected against neurotoxicity via the miR-34a/SIRT1/Beclin-1 pathway and activation of autophagy	Reduced 6-OHDA-induced neurotoxicity, enhanced autophagy, and improved neuronal survival	[230]
Theaflavin TF3	Theaflavin-3,3-digallate (TF3)	Hepatocytes	Preclinical–in vitro	In vitro experimental cell study	Reduced lipid deposition via inhibition of plasma kallikrein and activation of the AMPK signaling pathway	Decreased hepatocyte lipid droplet accumulation; proposed benefit for NAFLD-related lipid dysregulation	[231]
Thymoquinone		Isoproterenol-induced myocardial infarction rat model	Preclinical–in vivo	In vivo experimental animal study	Preserved cardiac mitochondrial DNA and exerted antioxidant, anti-inflammatory, and anti-apoptotic effects	Reduced serum cardiac injury markers, oxidative stress, inflammatory cytokines, apoptosis, fibrosis, and histopathologic damage; preserved cardiac mtDNA content	[232]
Wuzi Yanzong Pill	Diverse compounds	CPZ-induced demyelination animal model	Preclinical–in vivo	In vivo experimental animal study	Improved central nervous system (CNS) microenvironment by reducing neuroinflammation, modulating microglial phenotype, and increasing neurotrophic factors	Alleviated demyelination, improved mood-related behavior, and promoted remyelination	[233]
		Mouse model of Parkinson’s disease	Preclinical–in vivo pharmacology plus in vivo	In vitro and in vivo experimental study	Inhibits apoptosis and increases the secretion of neurotrophic factor via PI3K/AKT signaling pathway	Anti-apoptotic effect and modulation of neuronal survival	[233]

**Table 2:** Summary of the acute effects of antiaging bioactive molecules and herbal medicine therapies on the prevention of telomere shortening and damage, reduction in oxidative stress, as well as vascular senescence and inflammation; enhancement of neuroprotection; and improvement of plasma total antioxidant capacity.

Herb/Bioactive	Main Bioactive Compound	Study Subjects/Sample Size	Experimental Setting/Model	Study Design	Mechanism of Action	Pharmacological Effects	Ref.
Astragalus-based nutritional supplement	Cycloastragenol-rich Astragalus-based supplement	Middle-aged adults (n = 96)	Clinical trial	RCT (double-blind) placebo-controlled (6-month intervention)	Telomere-supporting effects, likely through telomerase activation/telomere maintenance	Increased telomere length compared with placebo after supplementation	[234]
*Aronia melanocarpa* supplement	Anthocyanin-rich aronia juice-based food supplement	91 healthy volunteers; ex vivo peripheral blood lymphocytes from a male subgroup	Clinical trial	RCT (8-week intervention)	DNA-protective antioxidant effects; reduced susceptibility of lymphocytes to oxidative DNA damage ex vivo	Reduced H_2_O_2_-induced DNA strand breaks ex vivo; decreased background DNA strand breaks compared with baseline	[235]
Five-edible-plant dietary supplement (Mylife/Mylife100^®^)	Multi-component edible plant extract blend from black sesame seed, guava fruit, mangosteen aril, pennywort leaves, and soy protein	Thai adults aged 50–65 years (n = 32)	Clinical trial	RCT (8-week intervention)	Telomere-supporting effects, likely related to antioxidant activity	Increased leukocyte telomere length and improved plasma total antioxidant capacity compared with placebo	[236]
Nicotinamide riboside		Older adults with mild cognitive impairment (n = 20)	Clinical trial	RCT (10-week intervention)	NAD+ precursor; supports cellular energetics and neuroprotective/geroscience-related pathways	Evaluated cognitive and aging-related outcomes in older adults with MCI	[237]
Pomegranate extract	Pomegranate polyphenol-rich extract	Older adults aged 55–70 years (n = 72 completers)	Clinical trial	RCT (12-week intervention)	Likely antioxidant and vascular-aging-related effects associated with modulation of IGF-1	Increased serum IGF-1 at week 12; no significant effect on telomere length	[238]
Quercetin		Symptomatic coronary artery disease patients undergoing coronary artery bypass graft surgery (n = 97)	Clinical trial	RCT (2 days pre-surgery)	Senolytic/anti-inflammaging effects; reversed vascular senescence and inflammatory signaling in male vascular cells and improved endothelial function ex vivo	Reduced vascular senescence and inflammation in symptomatic male but not female CAD patients; improved acetylcholine-induced endothelial relaxation in men; lowered postoperative atrial fibrillation incidence	[239]
Resveratrol		Older adults with type 2 diabetes (97 participants; 1000 mg/day group n = 37, 500 mg/day group n = 32, placebo n = 28)	Clinical trial	RCT (6-month intervention)	Antioxidant effects with modulation of oxidative-stress markers and SIRT1	Decreased lipoperoxides and carbonyl stress markers, increased total antioxidant capacity and SIRT1, but no significant change in glucose or HbA1c	[240]

## Data Availability

No new data were created or analyzed in this study.

## Abbreviations

The following abbreviations are used in this manuscript:

AD	Alzheimer’s disease;
AMPK	AMP-activated protein kinase;
Ang II	Angiotensin II;
AT1R	Angiotensin II type 1 receptor;
CVD	Cardiovascular disease;
CNS	Central nervous system;
DAG	Diacylglycerol;
DFI	DNA fragmentation index;
DNMT	DNA methyltransferase;
EGCG	Epigallocatechin gallate;
ER	Extracellular vesicle;
Foxo	Forkhead box O;
GPCRs	G protein-coupled receptors;
GSK3β	Glycogen synthase kinase 3 beta;
HDAC	Histone deacetylase;
HDL	High-density lipoprotein;
IGF-1	Insulin-like growth factor 1;
IGF-2	Insulin-like growth factor 2;
IP3	Inositol trisphosphate;
IRS-1	Insulin receptor substrate 1;
IRS-2	Insulin receptor substrate 2;
InsR	Insulin resistance;
IRs	Insulin receptors;
MAPK	Mitogen-activated protein kinase;
MASH	Metabolic dysfunction-associated steatohepatitis;
MASLD	Metabolic dysfunction-associated steatotic liver disease;
MetS	Metabolic syndrome;
MMPs	Matrix metalloproteinases;
mTOR	Mammalian target of rapamycin;
NO	Nitic oxide;
PBMCs	Peripheral blood mononuclear cells;
PLC	Phospholipase C;
PI3K	Phosphatidylinositol 3-kinase;
RAS	Renin–angiotensin system;
RCT	Randomized controlled trial;
ROS	Reactive oxygen species;
SASP	Senescence-associated secretory phenotype;
SIRT	Sirtuin;
SOD	Superoxide dismutase;
TBC1D4	TBC1 domain family member 4;
NF-κB	Transcription factor nuclear factor-κB;
TRP	Transient receptor potential;
TSC1	Tuberous sclerosis complex 1;
TSC2	Tuberous sclerosis complex 2;
T2DM	Type 2 diabetes mellitus;
TNF-α	Tumor necrosis factor–α;
UKPDS	United Kingdom Prospective Diabetes Study;
UN	United Nations.
